# Transcriptomics Provide Insights into the Photoperiodic Regulation of Reproductive Diapause in the Green Lacewing, *Chrysoperla nipponensis* (Okamoto) (Neuroptera: Chrysopidae)

**DOI:** 10.3390/insects15020136

**Published:** 2024-02-17

**Authors:** Shaoye Liu, Yuqing Gao, Rangjun Shi, Haiyi Huang, Yongyu Xu, Zhenzhen Chen

**Affiliations:** 1 College of Plant Protection, Shandong Agricultural University, Tai’an 271018, China; shaoyeliu2016@163.com (S.L.); 15550855190@163.com (Y.G.); 15078429100@163.com (H.H.); 2 State Key Laboratory of Integrated Management of Pest Insects and Rodents, Institute of Zoology, Chinese Academy of Sciences, Beijing 100101, China; shirangjun22@ioz.ac.cn

**Keywords:** photoperiod, diapause-sensitive stage, reproductive development, Jhamt

## Abstract

**Simple Summary:**

*Chrysoperla nipponensis* (Okamoto) is an important predatory insect, primarily preying on various agricultural pests such as aphids. The green lacewing exhibits facultative diapause, which is primarily influenced by the photoperiod. Although previous research has shown the physiological phenotypic responses of diapause-sensitive and pre-diapause periods to photoperiods in *C. nipponensis*, the corresponding regulatory mechanisms underlying its diapause remain somewhat unclear. In this study, transcriptome sequencing was performed to explore the molecular basis for diapause, and dsRNA-mediated knockdown was used to elucidate the role of juvenile hormone acid methyltransferase 1 (*Jhamt1*) in regulating diapause in *C. nipponensis*. Our findings not only provide insights into the transcriptional regulation processes of genes within diapause-sensitive and pre-diapause periods but also unveil the key genes responsible for regulating diapause in *C. nipponensis* during the pre-diapause phase.

**Abstract:**

*Chrysoperla nipponensis* (Okamoto) displays typical adult reproductive diapause under short photoperiods; however, our understanding of the molecular mechanism underlying photoperiod-sensitive reproduction remains limited. In this study, we performed transcriptome profiling of four treatments (the diapause-sensitive stage and pre-diapause phase under long and short photoperiods) of *C. nipponensis* using RNA sequencing (RNA-seq). A total of 71,654 unigenes were obtained from the samples. Enrichment analysis showed that fatty acid metabolism-related pathways were altered under a short photoperiod. Moreover, β-oxidation-related gene expression was active during the diapause-sensitive period under a short photoperiod. The knockdown of juvenile hormone acid methyltransferase 1 (*Jhamt1*) prolonged the pre-oviposition period but did not affect the reproductive ability of female individuals in *C. nipponensis*. These findings provided us with a more comprehensive understanding of the molecular mechanisms of photoperiod-sensitive diapause and show that groundwork is crucial for bolstering the long-term storage and biocontrol potential of *C. nipponensis*.

## 1. Introduction

Diapause is a physiological state in which an insect’s growth and development are stagnant, in order to adjust to a periodic adverse natural environment [1,2]. Multivoltine insect diapause is most commonly facultative, meaning that it is induced by environmental factors [3,4,5]. Environmental factors that cause insect diapause include photoperiods, temperature, and nutrition, with the most important being changes in photoperiods that induce insect diapause [6]. The onset of diapause in insects is often influenced by the hormonal levels within their bodies [7,8,9]. When diapause occurs in the adult stage, it is mainly the reproductive system that is significantly suppressed [10]. During the induction process of diapause in insects, notable changes in physiological characteristics and gene expression often occur. Diapause is sometimes accompanied by changes in body coloration; for example, the chalcid wasp *Tetrastichus septentrionalis* has two body colors, light yellow in the non-diapause state and gray-brown in the diapause state [11]. For instance, there is an initiation of yolk deposition, an accumulation of lipids in the fat body, and the activation of genes involved in fatty acid synthesis [12,13,14]. Meanwhile, trehalose can also be used as a protective agent in insect diapause, and in the wheat blossom midge *Sitodiplosis mosellana*, trehalose levels increase before diapause, peak during the diapause maintenance period, and decrease significantly after diapause is terminated [15]. During the pre-diapause phase, hormonal changes occur within the insect’s body, exerting a significant impact on the subsequent progression of diapause [12]. Juvenile hormone (JH) is one of the most important hormones in insects and regulates many aspects of insect physiology, such as reproduction, diapause, development, and morphology maintenance [16,17,18]. The function of the key rate-limiting enzyme juvenile hormone acid methyltransferase (Jhamt) in the synthesis process of juvenile hormones has been identified in multiple species. Following the knockout of *Jhamt*, it is often accompanied by a decrease in juvenile hormone (JH) levels, the delayed development of the reproductive system, and a reduced rate of fat consumption [19,20]. Additionally, the degradation of juvenile hormone (JH) is involved in regulating diapause. In species such as the Cabbage beetle *Colaphellus bowringi*, the Asian lady beetle *Harmonia axyridis*, and the ladybird beetle *Coccinella Septempunctata*, knocking down JH degradation-related genes in diapausing individuals inhibits fat storage and suppresses the expression of stress tolerance genes and vitellogenin genes [21,22,23]. 20-hydroxyecdysone (20E) can influence JH synthesis through direct or indirect mechanisms in the mosquito *Aedes aegypti* [24], the fly *Drosophila melanogaster* [25], and *C. bowringi* [26]. Furthermore, prior research has demonstrated the involvement of the insulin signaling (IS) pathway in regulating diapause for several insect species [27,28,29,30]. The in vitro cultivation of *A. aegypti* Corpora allata–corpora cardiaca complexes (CA-CC), when treated with insulin, resulted in a 2–3 fold increase in JH synthesis in [31]. In the majority of studies, there has been a predominant focus on investigating the mechanisms underlying gene regulation during reproductive diapause. However, research pertaining to the sensitive period of diapause remains relatively scarce.

Green lacewings, *Chrysoperla nipponensis* (Okamoto) are an ecologically important group of predatory insects in nature. They primarily prey on species that are often pests, such as aphids, thrips, whiteflies, and lepidopteran larvae. They are widely distributed in the major rice- and wheat-producing regions of China. The larvae of *C. nipponensis* are known for their significant predation on aphids, making them excellent natural predators for pest control, particularly during the spring season [32,33,34]. *C. nipponensis* undergoes multiple generations within a year, although variations exist among different geographic populations. During the spring and autumn seasons, the average lifespan of the female adult stage is typically around 50 to 60 days, while during the summer, it is approximately 30 to 40 days. Following the conclusion of October, the predominant presence comprises overwintering adults, which disperse themselves among late-maturing crops that serve as hosts for aphids or embark on a migration toward designated wintering habitats. *C. nipponensis* is a holometabolous insect species. It undergoes complete metamorphosis, consisting of three larval instars. *C. nipponensis* exhibits facultative diapause in adulthood, wherein adults enter a diapause state under short photoperiods and a non-diapause state (reproductive state) under long photoperiods. During adult diapause, their body color changes from green to tan or yellowish, accompanied by the appearance of diapause spots. Based on our previous observations, we have found that starting on the fifth day after adult emergence, diapausing and non-diapausing female adults begin to exhibit differences in body color and weight [35]. Although *C. nipponensis* can sense short photoperiods and prepare for diapause upon adult eclosion, early laboratory studies revealed that experiencing short photoperiods during the larval stage also affects the physiological state of individuals [36]. Compared with long photoperiods, a short photoperiod significantly extends the developmental durations of the first and second instars. In contrast, the third instar and prepupal stage show no such difference between these two photoperiodic conditions [36]. Additionally, it is noteworthy that, in third-instar larvae, cold-induced mortality rates during the developmental process are significantly lower under short photoperiods compared with long photoperiods [37]. This implies that the photoperiod not only causes physiological changes in adults but also has potential effects on larvae. Environmental factors inducing diapause have been widely studied, but there are few reports on the sensitive period of diapause induction [10,11,38]. It has been observed that third-instar larvae and pre-pupae exhibit greater sensitivity compared with other stages of preimaginal development in *C. nipponensis* [32]. Moreover, their effects on the pre-oviposition period of the female adult stage—upon transitioning into a prolonged photoperiod during three developmental periods (third instar, pre-pupae, and pupae)—have been found to have a cumulative impact [32]. Therefore, the 3rd instar larval stage marks the initial crucial period during the larval phase that impacts the pre-oviposition period of the female adult stage [32].

In this study, we conducted an investigation of the transcriptomic mechanisms underlying the photoperiod-sensitive female-adult-stage diapause in *C. nipponensis*. This was carried out to enhance our comprehension of the phenotypic aspects investigated in previous physiological and biochemical studies, as well as to further elucidate how photoperiods regulate reproduction in female adults. These findings have provided us with a more comprehensive insight into the photoperiod-sensitive period of diapause induction.

## 2. Materials and Methods

### 2.1. Experimental Insects

*C. nipponensis* adults were collected from a plant nursery in Tai’an, Shandong Province, China (36°15′ N, 116°59′ E). Adults were paired in glass cylinders (18 cm high, 9 cm in diameter) and supplied with a dry powdered mixture of yeast–sugar (yeast: sugar = 10: 8) and a 10% honey–water solution as food. The cylinders were kept in an environmental chamber (RSZ Artificial Intelligence Phytotron, Changzhou, China) set at a photoperiod of 15:9, with a constant 25 °C temperature and 60% humidity.

Eggs were collected daily, and the larvae were reared individually on *Aphis craccivora* Koch in glass tubes (2 cm in diameter, 7 cm in length) under the above conditions. After two generations, eggs were randomly divided into two groups for transcriptome sequencing. The diapause of *C. nipponensis* is photoperiod-dependent, with short photoperiods inducing adult diapause and long photoperiods inducing non-diapause in adults. The diapause larvae and adults used in this study were reared at 25 °C under short-day photoperiodic conditions (9:15 h (L:D)) during their development, while non-diapause individuals were maintained at 25 °C under long-day photoperiodic conditions 15:9 h (L:D). Four conditions of *C. nipponensis* were collected, including diapause 3rd-instar larvae at day 3 (S_larvae), non-diapause 3rd-instar larvae at day 3 (L_larvae), diapause female adults at day 5 after eclosion (S_Female), and non-diapause female adults at day 5 after eclosion (L_Female).

Three independent biological replicates were performed for each group. All samples were frozen in liquid nitrogen upon collection and stored at −80 °C for the following transcriptomic analyses.

### 2.2. RNA Extraction

Total RNA was extracted from the whole body using TRIzol^®^ Reagent (Invitrogen, Carlsbad, CA, USA). Then, RNA quality was determined by a 5300 Bioanalyser (Agilent, Santa Clara, CA, NA, USA) and quantified using the ND-2000 (NanoDrop Technologies, Carlsbad, CA, USA). A high-quality RNA sample (OD260/280 = 1.8~2.2, OD260/230 ≥ 2.0, RIN ≥ 6.5, 28S:18S ≥ 1.0, >1 μg) was used to construct a sequencing library.

### 2.3. Illumina RNA-seq Library Construction and Sequencing

RNA purification, reverse transcription, library construction, and sequencing were conducted at Shanghai Majorbio Bio-pharm Biotechnology Co., Ltd. (Shanghai, China), following the manufacturer’s instructions (Illumina, San Diego, CA, USA). The *C. nipponensis* RNA-seq transcriptome library was prepared using 1 μg of total RNA with Illumina^®^ Stranded mRNA Prep Ligation (San Diego, CA, USA). Messenger RNA was isolated via the polyA selection method with oligo (dT) beads and then fragmented using a fragmentation buffer. Double-stranded cDNA was synthesized using a SuperScript double-stranded cDNA synthesis kit (Invitrogen, Carlsbad, CA, USA) with random hexamer primers (Illumina). The synthesized cDNA was subjected to end-repair, phosphorylation, and ‘A’ base addition following Illumina’s library construction protocol. The libraries were size-selected for cDNA target fragments of 300 bp on 2% Low Range Ultra Agarose and amplified via PCR using Phusion DNA polymerase (NEB) for 15 PCR cycles. After quantification using Qubit 4.0, the paired-end RNA-seq sequencing library was sequenced using the NovaSeq 6000 sequencer (2 × 150 bp read length).

### 2.4. Illumina Data Analysis

The raw paired-end reads were trimmed and quality controlled by fastp (v0.19.5) [39] with default parameters. Then, clean data from the samples (S_larvae, L_larvae, S_Female, L_Female) were used to perform de novo assembly with Trinity (v2.8.5) [40]. To increase the assembly quality, all the assembled sequences were filtered by CD-Hit (v4.5.7) and transrate (v1.0.3). The assembled transcripts were searched against the NCBI protein nonredundant (NR), COG, and KEGG databases using Diamond (v0.9.24) to identify the proteins that had the highest sequence similarity with the given transcripts to retrieve their function annotations, and a typical cut-off E-value of less than 1 × 10^−5^ was set. Metabolic pathway analysis was performed using the Kyoto Encyclopedia of Genes and Genomes (KEGG) [41].

### 2.5. KEGG Enrichment Analysis of Differentially Expressed Genes

To identify DEGs (differentially expressed genes) between two different samples, the expression level of each transcript was calculated according to the transcripts per million reads (TPM) method. RSEM [42] was used to quantify gene abundances. Essentially, differential expression analysis was performed using DESeq2 (v1.24.0) [43]. DEGs with |log2FC| ≥ 1 and FDR ≤ 0.05 were considered to be significantly differently expressed genes. In addition, KEGG functional enrichment analysis was performed to identify the significantly enriched pathways at a Bonferroni-corrected *p*-value ≤ 0.05 compared with the whole-transcriptome background. KEGG pathway analysis was carried out with KOBAS [44].

### 2.6. Real-Time Quantitative PCR

*Tub1* was used as a reference gene for quantitative RT-PCR [45]. Nineteen genes were selected from the transcriptome data for validation via qPCR (Figure S1). The Premier 5.0 software (Premier Biosoft, Palo Alto, CA, USA) was used to design the primers, and 80–160 bp products were selected (Table S10). Three biological replicates were evaluated for each gene qPCR system.

DNase-treated RNA (1 μg) was reverse-transcribed in a 20 µL reaction. Reverse transcription was performed in a 20 µL reaction mixture using a cDNA Reverse Transcription Kit (HiScript1l Q RT SuperMix for gPCR, R223-01, Vazyme, Nanjing, China). The 20 µL total reaction volume was configured according to the protocol of the ChamQ SYBR qPCR Master Mix (Q311-02, Vazyme, Nanjing, China), which contained 10 µL of 2 × ChamQ SYBR qPCR Master Mix, 0.4 µL (10 μM) of each gene-specific primer, 1 µL of cDNA, and 8.2 µL of ddH_2_O. Then, PCR reactions were performed using the CFX96 instrument (Bio-Rad, Hercules, CA, NA, USA). The amplification reaction program was set as follows: pre-denaturation at 95 °C for 30 s, followed by 40 cycles of denaturation at 95 °C for 10 s and annealing at 60 °C for 30 s. The melting curves were analyzed in the 60–95 °C temperature range after the amplification step. The resulting CT value of each gene was normalized to the geometric mean of the CT value of the reference gene (*Tub1*) by subtracting the mean CT of the reference gene from the CT value of the gene (2^−∆CT^ method).

### 2.7. RNA Interference

*CnJhamt1* and green fluorescent protein (GFP) primers were designed (Supplementary Materials). *dsGFP* was used as a control, while *dsJhamt1* was used as the treatment. The T7 RiboMAX^TM^ Express RNAi System (Promega, Madison, WI, USA) was employed for synthesizing dsRNA following the protocol. The concentration of dsRNA was measured using a NanoDrop One spectrophotometer (Thermo Scientific, Wilmington, DE, USA) and adjusted to 0.5 μg/μL. On the first day after adult eclosion, dsRNA was injected into each *C. nipponensis* individual using an Eppendorf FemtoJet^®^ 4i and an InjectMan 4 microinjector at a volume of 0.2 μL.

### 2.8. Statistical Analysis

The data were analyzed using the SPSS Statistics version 21 software (SPSS Inc., Chicago, IL, USA). The data are presented as mean ± s.e.m. Student’s *t*-test (*p* < 0.05) was used to determine differences between the pre-oviposition period, oviposition duration, number of eggs laid per female, and female longevity. Heat maps were crafted utilizing the TBTools v2.019 software. Figures were produced in the GraphPad Prism 8.0 software (GraphPad Software Inc., San Diego, CA, USA).

## 3. Results

### 3.1. Transcriptome Analysis of Larva and Female Adults Reared under a Short Photoperiod and a Long Photoperiod

**Figure 1:**
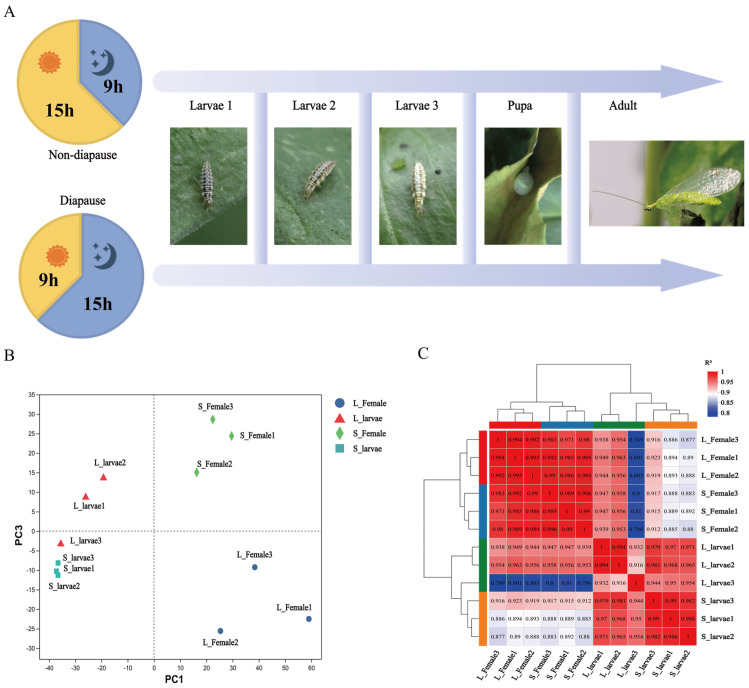
Overall transcriptome analysis of larvae and female adults reared under a short photoperiod and a long photoperiod in *C. nipponensis*. (**A**) Schematic diagram of sample collections for RNA−seq: larva reared during either a short photoperiod (L:D = 9:15 h) or a long photoperiod (L:D = 15:9 h) were collected at day 3 of the third instar; female adults reared during either a short photoperiod (L:D = 9:15 h) or a long photoperiod (L:D = 15:9 h) were collected at day 5 of adulthood. (**B**) Principal component analysis (PCA) of all 12 samples from the larval and female adult stages under different photoperiodic conditions. (**C**) Cluster analysis of all 12 samples from larval and female adult stages under different photoperiods. Larvae 1: 1st−instar larvae; Larvae 2: 2nd−instar larvae; Larvae 3: 3rd−instar larvae.

In this study, we selected and collected samples from four conditions to understand photoperiod-induced effects on *C. nipponensis* at different development stages. We performed RNAseq of the third-instar larvae and the female adult stage of the green lacewing (Figure 1A). A total of 71,654 unigenes and 104,173 transcripts were identified (Table S1). Principal component analysis (PCA) showed that the overall expression patterns were clearly separated between the female adult stage and larvae groups. Meanwhile, female adults reared under a long photoperiod were obviously distinguished from those reared under a short photoperiod (Figure 1B). Through a correlation analysis of the samples, it was found that, except for L_larvae3, the other samples at the same stage had high repeatability (Figure 1C).

### 3.2. Transcriptome Undergoes Changes during the Developmental Process

**Figure 2:**
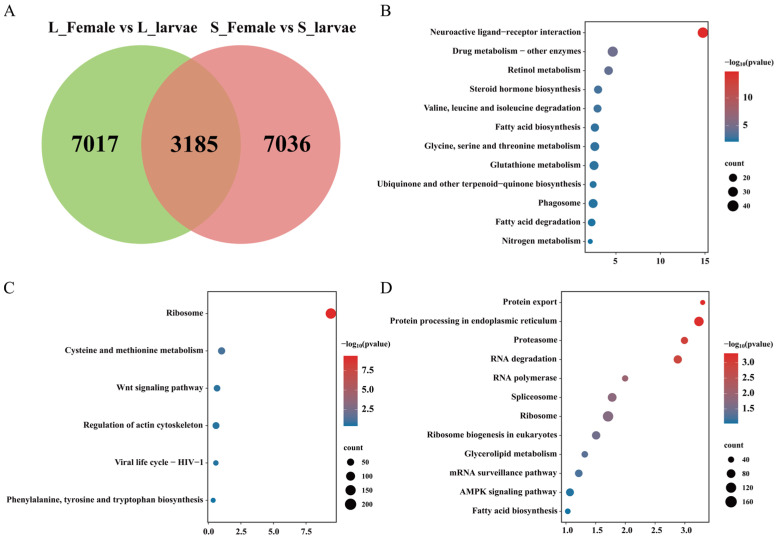
Comparison of gene expression patterns between female adults and larvae under different photoperiodic conditions in *C. nipponensis*. (**A**) Venn diagram indicating the number of differentially expressed genes (DEGs) in the transcriptome comparison between female adults and larvae under a short photoperiod and a long photoperiod. (**B**) KEGG enrichment analysis of 3185 shared DEGs in the L_Female vs. S_Female group and S_Female vs. S_larvae group. (**C**) KEGG enrichment analysis of 7017 DEGs in the L_Female vs. L_larvae group. (**D**) KEGG enrichment analysis of 7036 DEGs in the S_Female vs. S_larvae group. L_Female, long-photoperiod female; L_larvae, long-photoperiod larvae; S_Female, short-photoperiod female; S_larvae, short-photoperiod larvae; vs., versus.

Overall, there were 10,202 DEGs (6790 upregulated and 3412 downregulated) and 10,221 DEGs (7564 upregulated and 2653 downregulated) in the L_Female vs. L_larvae group and the S_Female vs. S_larvae group, respectively (Table S2). A total of 7017 development-related genes were found to respond exclusively to a long photoperiod, while 7036 genes were specifically responsive to the developmental process under a short photoperiod (Figure 2A). There were 3185 common DEGs that exhibited altered expression levels between the larval and female adult stages under two different photoperiodic conditions. These genes were subjected to KEGG analysis, and the results revealed significant enrichment in pathways, such as neuroactive ligand–receptor interactions, drug metabolism–other enzymes, retinol metabolism, and steroid hormone biosynthesis (Figure 2B and Table S3). A KEGG enrichment analysis of 7017 differentially expressed genes (DEGs) specifically between the L_Female and L_larvae groups was conducted. These DEGs primarily enriched ribosome, cysteine, and methionine metabolism and the Wnt signaling pathway (Figure 2C and Table S4). The 7036 development-related DEG genes that responded to short photoperiod conditions were enriched in pathways such as protein export, protein processing in the endoplasmic reticulum, and proteasome (Figure 2D and Table S5).

### 3.3. Transcriptome Undergoes Changes in Different Photoperiods

**Figure 3:**
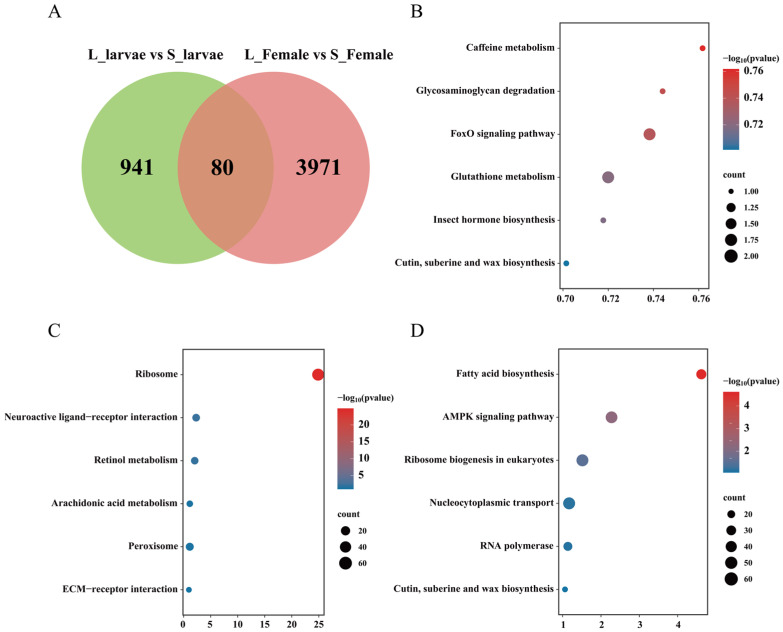
Comparison of gene expression patterns between long photoperiods and short photoperiods for different development stages of *C. nipponensis*. (**A**) Venn diagram indicating the number of DEGs in the L_larvae vs. S_larvae and L_Female vs. S_Female groups. (**B**) KEGG enrichment analysis of 80 shared DEGs in the L_larvae vs. S_larvae and L_Female vs. S_Female groups. (**C**) KEGG enrichment analysis of 941 DEGs in the L_larvae vs. S_larvae group. (**D**) KEGG enrichment analysis of 3971 DEGs in the L_Female vs. S_Female group.

In the comparison between the L_larvae and S_larvae groups, a total of 1021 differentially expressed genes (DEGs) were identified (consisting of 717 upregulated genes and 304 downregulated genes). In the L_Female vs. S_Female comparison, a higher number of DEGs was observed, with a total of 4051 DEGs identified, including 3465 upregulated genes and 586 downregulated genes (Table S2). Furthermore, among the identified DEGs, 80 genes exhibited responsive expression to photoperiods in both the larval and female adult stages, while 941 genes specifically responded to different photoperiods in larvae, and 3971 genes specifically responded to photoperiodic changes in the female adult stage (Figure 3A). Among these DEGs, 80 genes showed significant enrichment in pathways including caffeine metabolism; glycosaminoglycan degradation; the FoxO signaling pathway; glutathione metabolism; insect hormone biosynthesis; and cutin, suberine, and wax biosynthesis (Figure 3B and Table S6). In the L_larvae vs. S_larvae group, 941 DEGs were significantly enriched in pathways such as ribosome, neuroactive ligand–receptor interactions, and retinol metabolism (Figure 3C and Table S7). Furthermore, 3971 genes were enriched in pathways such as fatty acid biosynthesis, the AMPK signaling pathway, ribosome biogenesis in eukaryotes, and nucleocytoplasmic transport (Figure 3D and Table S8).

### 3.4. Expression Analysis of Multiple Metabolic Pathways

**Figure 4:**
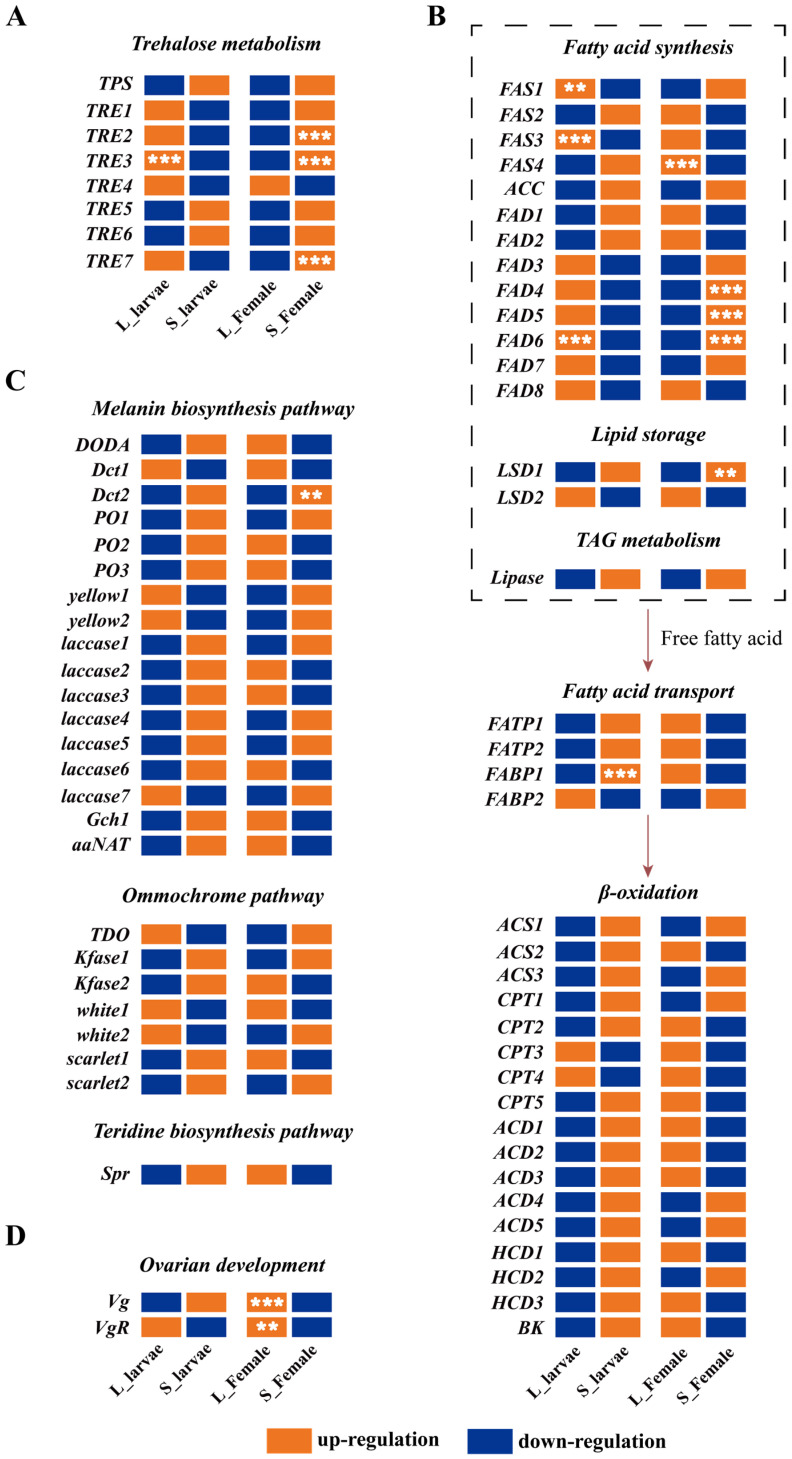
Expression analysis of genes related to trehalose metabolism, fatty acid metabolism, pigment biosynthetic pathways, and ovarian development genes between the female adults and larvae under a long photoperiod. (**A**) Heat map of 8 differentially expressed genes associated with trehalose metabolism. (**B**) Heat map of 37 differentially expressed genes associated with fatty acid metabolism. (**C**) Heat map of 25 differentially expressed genes associated with pigment biosynthetic pathways. (**D**) Heat map of 2 differentially expressed genes associated with ovarian development. ** *p* < 0.01; *** *p* < 0.001. The color orange signifies upregulated genes under varying photoperiods within the same developmental stage, while the color blue represents downregulated genes under similar photoperiods within the identical developmental stage.

The expression patterns of *TRE2* and *TRE7* exhibited similarities, with higher expression observed at the female adult stage. Interestingly, the expression of *TRE3* was found to be upregulated in the larval long-photoperiod state, whereas the gene displayed upregulation during the female adult stage short photoperiod (Figure 4A). The expression patterns of genes related to the fatty acid synthesis pathway underwent significant changes across different developmental stages. The expression of genes involved in fatty acid synthesis, including *FAS1*, *FAS3*, and *FAS4*, showed higher transcript levels in L_larvae. On the other hand, the transcript levels of *FAD3*, *FAD4*, *FAD5*, *FAD6*, and *FAD7* were found to be higher in the S_Female group (Figure 4B). Transcription levels of *FATP1*, *FATP2*, *FABP1*, *ACS2*, *CPT2*, *CPT5*, *ACD1*, *ACD2*, *ACD3*, *HCD1*, *HCD3*, and *BK* were observed to increase in both the S_larvae and L_Female states (Figure 4B). In the melanin biosynthesis pathway, only the expression of *Dct2* showed a significant upregulation in the S_Female group (Figure 4C). The transcription levels of *Vg* and *VgR*, genes associated with reproduction, were found to be highest in the L_Female group (Figure 4D).

### 3.5. Hormone Gene Expression Profile in the Green Lacewing

**Figure 5:**
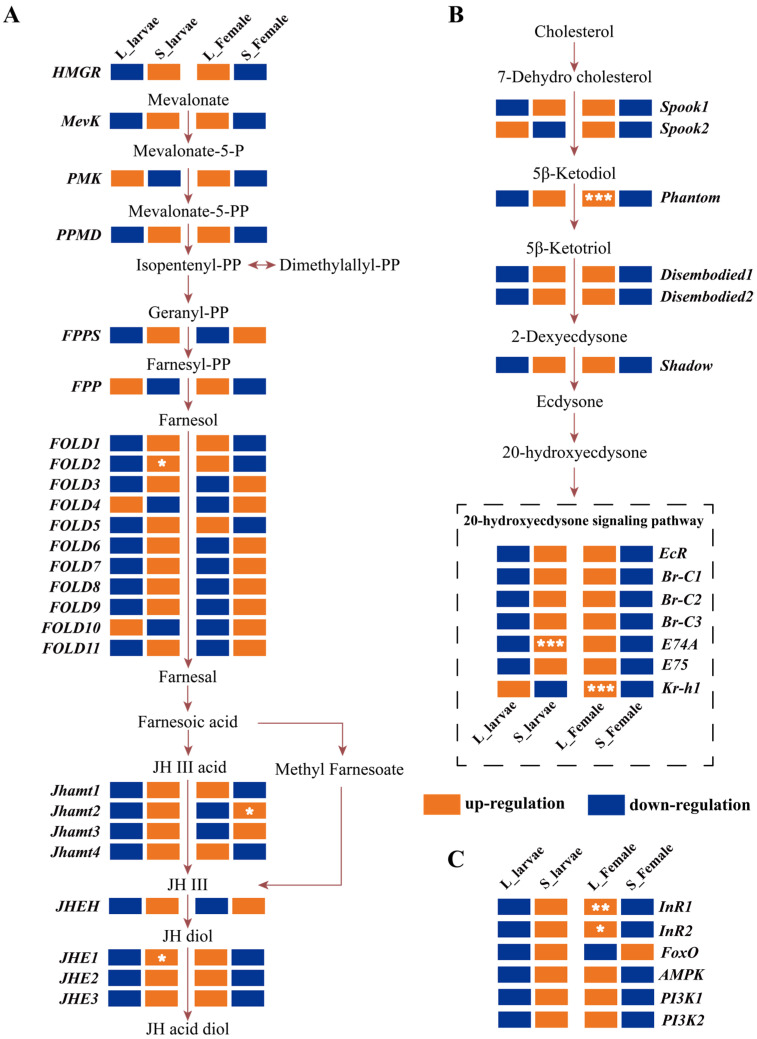
Expression analysis of genes involved in JHIII, 20-hydroxyecdysone, and insulin pathways between female adults and larvae under a long photoperiod. (**A**) JHIII signaling pathway. (**B**) Summary of 20-hydroxyecdysone pathway. (**C**) Insulin pathway. * *p* < 0.05; ** *p* < 0.01; *** *p* < 0.001. Under different photoperiodic conditions, genes are either upregulated or downregulated within the same developmental stage. The color orange represents genes that are upregulated under different photoperiods within the identical developmental stage, while the color blue indicates genes that are downregulated under different photoperiods within the same developmental stage.

During the third-instar larvae, the transcription levels of most genes involved in juvenile hormone synthesis and degradation pathways were upregulated under the short photoperiod. Specifically, *FOLD2* and *JHE3* exhibited significant upregulation. However, during the female adult stage, the majority of gene transcription was primarily upregulated under long photoperiod (Figure 5A). Interestingly, *FPPS*, most *FOLD* genes (*FOLD3*, *FOLD4*, *FOLD6*, *FOLD7*, *FOLD8*, *FOLD9*, *FOLD10*, *FOLD11*), *Jhamt1*, *Jhamt2*, and *JHEH* displayed continued upregulation in their transcription levels under the short photoperiod (Figure 5A). In the 20E signaling pathway, most genes exhibited expression patterns similar to the juvenile hormone pathway. They showed an upregulation in gene expression levels during the larval stage under the short photoperiod and during the female adult stage under the long photoperiod (Figure 5B). In the insulin signaling pathway specifically, *InR1* and *InR2* demonstrated significant upregulation exclusively during the female adult stage under the long photoperiod (Figure 5C).

### 3.6. Functional Validation of Jhamt1 in Pre-Diapause Phase

**Figure 6:**
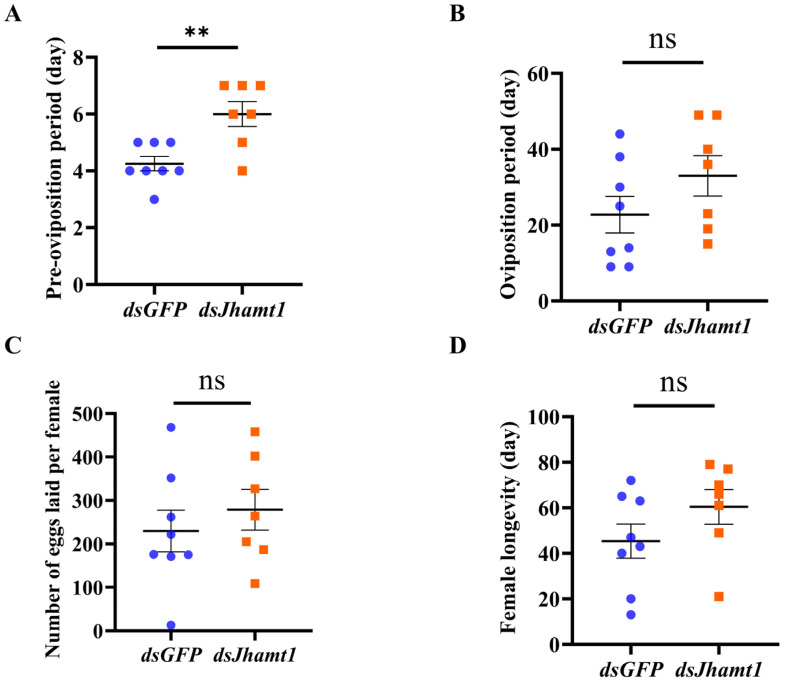
Effects of *Jhamt1* knockdown on fecundity and longevity in *C. nipponensis*. The pre-oviposition period after *Jhamt1* gene knockdown (**A**), oviposition duration (**B**), number of eggs laid per female (**C**), and female longevity (**D**) of green lacewing non-diapause female adults injected with *dsJhamt1* on the first day of eclosion. The double asterisks indicate extremely significant differences (*p* < 0.01) in various indexes of female adults subjected to and not subjected to *dsJhamt1* treatment at one day of age, using a topical application (*t*-test); ns, no significance.

Given the disparate expression patterns of *Jhamt1* in the larval and female adult stages, as well as substantial variations in gene expression between different photoperiods, it is imperative to further elucidate the functionality of *Jhamt1*. Consequently, we conducted a *Jhamt1* knockdown intervention during the pre-diapause phase and subsequently documented discernible alterations in the reproductive capacity of the female adult stage. In this study, we found a significant extension of the pre-oviposition period (Figure 6A). We also examined oviposition duration (Figure 6B), the number of eggs laid per female (Figure 6C), and female longevity (Figure 6D) as indicators, but no significant differences were observed between the *dsGFP* and *dsJhamt1* treatments.

## 4. Discussion

Under a long photoperiod, *C. nipponensis*, a holometabolous insect, undergoes metamorphosis from larva to adult, including a pupal stage, which requires significant protein resynthesis (Figure 2B). Ribosomes are a critical component involved in cellular protein synthesis [46,47]. Protein processing and fatty acid biosynthesis pathways exclusively respond to the developmental process under a short photoperiod (Figure 2D). Moreover, in the diapause state of *C. septempunctata*, there are also changes in genes related to the fatty acid biosynthesis pathway [12]. This implies that the synthesis of lipid substances plays a crucial role in the development of *C. nipponensis* under short photoperiods.

As mentioned earlier, in diapause insects, there is an increase in lipid substances and a suppression of lipid metabolism [3,12]. Our transcriptomic results show that the expression levels of *FAD4*, *FAD5*, *FAD6*, and *LSD1* are elevated in adult females under a short photoperiod (Figure 4B). Interestingly, the transcription levels of genes associated with lipid storage did not show a significant increase under a short photoperiod in larvae. Instead, the majority of genes involved in lipid metabolism processes were upregulated (Figure 4B). This implies that larvae under a short photoperiod may generate more ATP, resulting in a significantly lower mortality rate when exposed to low-temperature treatments compared with those under long photoperiods. This suggests that diapause and non-diapause states may employ distinct strategies in response to changes in photoperiods.

Body color switch is a common strategy employed by some insects to respond to external signals and enhance their adaptability, for example, locusts, silkworm larvae, and southern green stink bugs [48,49,50,51]. During the transition from non-diapause to diapause in *C. nipponensis*, there is a change in the ventral color from green to tan and a change in the dorsal diapause patch from brown to reddish-brown [52]. Transcriptome analysis revealed that, besides *Dct2*, there were no significant changes in the transcription levels of other genes related to pigment synthesis pathways in the S_Female group. Perhaps because of the experimental treatments being conducted in the early stages of diapause induction, there was a lack of pronounced phenotypic differences between the two photoperiod treatments. However, the differential expression of *Dct2* may imply the initiation of body color change within *C. nipponensis* under diapause-inducing conditions (Figure 4C).

The regulatory role of juvenile hormones in insects is of great importance, and there is substantial evidence that changes in juvenile hormone titers can impact insect reproduction and diapause [8,18]. Furthermore, the expression of genes related to 20E, the insulin pathway, juvenile hormone synthesis, and degradation can also regulate juvenile hormone levels [19,22,24,31]. In the transcriptomic analysis, we observed higher expression levels of *Jhamt1*, *Jhamt2*, *Jhamt3*, *Jhamt4*, *JHEH*, *JHE1*, *JHE2*, and *JHE3* in S_larvae compared with L_larvae (Figure 5A). In previous studies, it was found that, under short photoperiods, the developmental duration of the first instar and the second instar is significantly longer than under a long photoperiod, but there is no significant difference in the developmental duration of the third instar [36]. Under a short photoperiod, the tolerance of third-instar larvae to low temperatures is significantly higher than under a long photoperiod [37]. This intriguing finding further implies a potential correlation between alterations in the transcriptional levels of juvenile hormone-related genes and the regulation of cold tolerance in third-instar larvae.

In our recent study, we found that exogenous JH treatment significantly shortened the pre-oviposition period of diapausing *C. nipponensis* female adults [53]. A similar result has also been observed in the oriental armyworm *Mythimna separata* and *C. septempunctata* [54,55,56]. Here, we knocked down the key gene *CnJhamt1*, involved in juvenile hormone synthesis in diapause-sensitive *C. nipponensis* during the diapause-sensitive period, and we found that the pre-oviposition period of *C. nipponensis* was significantly prolonged (Figure 6A), which indicated that the gene has an influence on the reproductive capacity of diapause-sensitive *C. nipponensis*. However, it did not affect oviposition duration (Figure 6B), the number of eggs laid per female (Figure 6C), or female longevity (Figure 6D). This suggested that early *CnJhamt1* treatment can delay the pre-oviposition period without compromising the reproductive capacity of individuals. Our findings are of great significance for extending the shelf life of the natural enemies of insect pests.

In summary, we used RNA-seq to analyze gene expression changes during the photoperiod-sensitive stage and pre-diapause phase in *C. nipponensis*. The high expression of β-oxidation-related genes in the S_larvae group increased the cold tolerance of the larvae. An RNAi experiment targeting the key gene *CnJhamt1*, which affects insect diapause, provides a theoretical basis for the further analysis of the diapause regulation mechanism in *C. nipponensis*.

## Data Availability

The raw data can be accessed on the NCBI website (https://www.ncbi.nlm.nih.gov/sra/PRJNA1057078, accessed on 1 March 2023) using the BioProject ID PRJNA1057078.

## Supplementary Materials

The following supporting information can be downloaded at https://www.mdpi.com/article/10.3390/insects15020136/s1: Figure S1: Validation of gene expressions by RT-qPCR. Table S1: Assembly result evaluation table. Table S2: Table of differential gene counts. Table S3: Summary of the KEGG pathways involved in 3185 DEGs. Table S4: Summary of the KEGG pathways involved in 7017 DEGs. Table S5: Summary of the KEGG pathways involved in 7036 DEGs. Table S6: Summary of the KEGG pathways involved in 80 DEGs. Table S7: Summary of the KEGG pathways involved in 941 DEGs. Table S8: Summary of the KEGG pathways involved in 3971 DEGs. Table S9: Genetic pathways and expression levels TPM. Table S10: Major primer sequences in this study.
