# Supplementary material for: Transcriptomics Provide Insights into the Photoperiodic Regulation of Reproductive Diapause in the Green Lacewing, Chrysoperla nipponensis (Okamoto) (Neuroptera: Chrysopidae)
Source: Insects. 2024 Feb 17;15(2):136. doi: 10.3390/insects15020136 (PMC10889211; doi:10.3390/insects15020136)
Supplement: Supplementary file 1 [file insects-15-00136-s001.zip › insects-2801740-supplementary.pdf]

Figure S1 Validation of gene expressions by RT-qPCR.

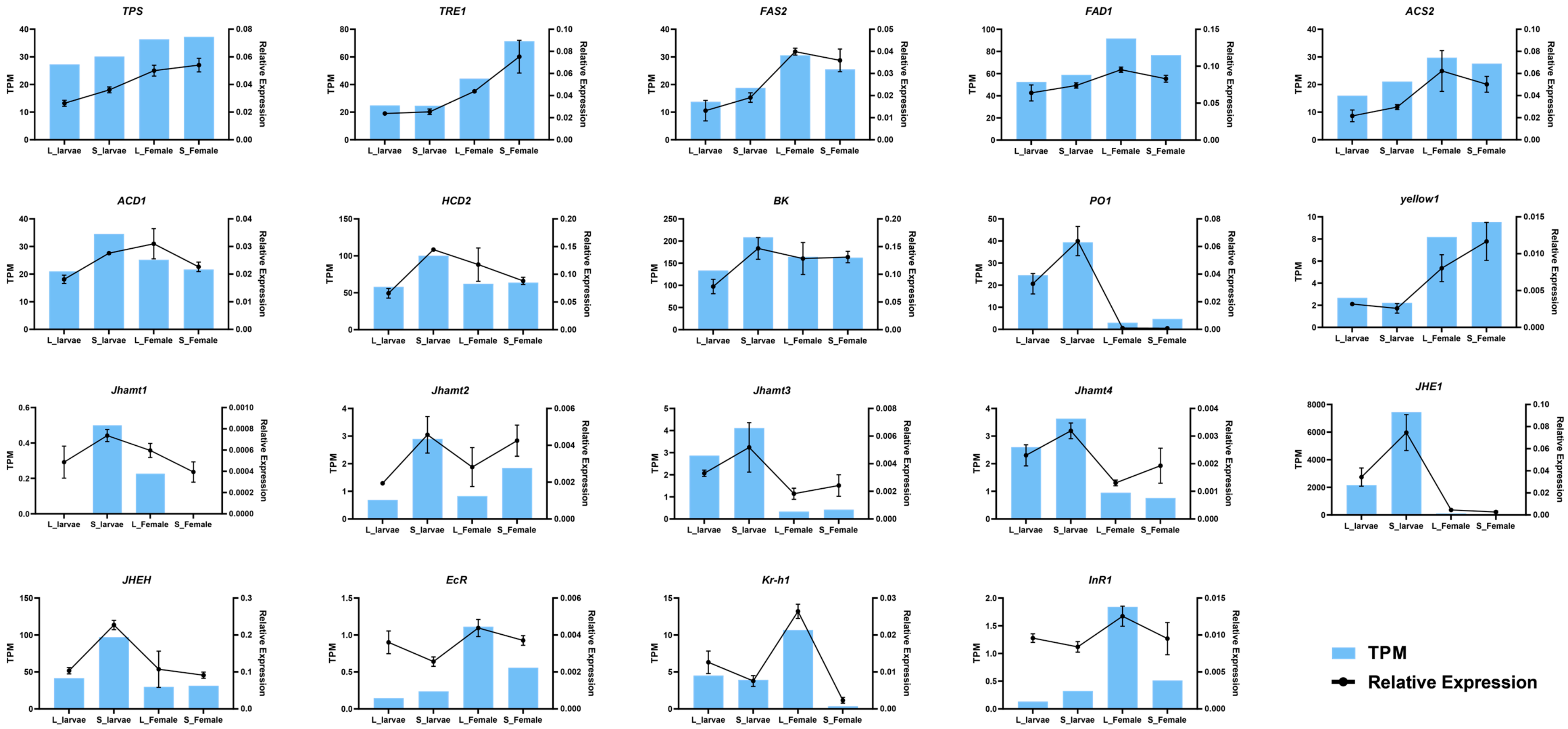

Table S1 Assembly result evaluation table

Type	Unigene	Transcript
Total number	71654	104173
Total base	68548641	106753458
Largest length (bp)	19152	19152
Smallest length (bp)	201	201
Average length (bp)	956.66	1024.77
N50 length (bp)	1637	1725
E90N50 length (bp)	2335	2174
Fragment mapped percent(%)	75.205	82.957
GC percent (%)	35.84	35.26
TransRate score	0.38171	0.41304
BUSCO score	C: 98.0% [S:90.9%; D:7.1%]	C: 98.4% [S: 68%; D: 30.4%]

Table S2 Table of Differential Gene Counts

Different Group	Total DEG	Up	Down
L_Female_vs_L_larvae	10202	6790	3412
S_Female_vs_S_larvae	10221	7568	2653
L_larvae_vs_S_larvae	1021	717	304
L_Female_vs_S_Female	4051	3465	586

Table S3 Summary of the KEGG pathways involved in 3185 DEGs

term	enrichment	pvalue	count
Neuroactive ligand-receptor interaction	14.76985812	1.70E-15	44
Drug metabolism - other enzymes	4.639324619	2.29E-05	41
Retinol metabolism	4.173024222	6.71E-05	26
Steroid hormone biosynthesis	2.993066064	0.001016094	22
Valine, leucine and isoleucine degradation	2.93113727	0.001171825	21
Fatty acid biosynthesis	2.634109739	0.00232215	23
Glycine, serine and threonine metabolism	2.62558966	0.002368156	28
Glutathione metabolism	2.53920285	0.00288933	29
Ubiquinone and other terpenoid-quinone biosynthesis	2.436576808	0.003659512	15
Phagosome	2.434558804	0.003676556	29
Fatty acid degradation	2.272192254	0.005343278	19
Nitrogen metabolism	2.115250699	0.007669187	11

Table S4 Summary of the KEGG pathways involved in 7017 DEGs

term	enrichment	pvalue	count
Ribosome	9.404759173	3.94E-10	210
Cysteine and methionine metabolism	1.019239825	0.095666564	54
Wnt signaling pathway	0.669852744	0.213868713	39
Regulation of actin cytoskeleton	0.591049579	0.256419129	47
Viral life cycle - HIV-1	0.579490273	0.263335693	24
Phenylalanine, tyrosine and tryptophan biosynthesis	0.373230732	0.423417952	23

Table S5 Summary of the KEGG pathways involved in 7036 DEGs

term	enrichment	pvalue	count
Protein export	3.300505866	0.000500604	33
Protein processing in endoplasmic reticulum	3.237569082	0.00057867	121
Proteasome	2.993915258	0.001014109	54
RNA degradation	2.881784908	0.00131285	81
RNA polymerase	1.995610531	0.010101584	40
Spliceosome	1.777712998	0.016683494	96
Ribosome	1.708541557	0.019564036	163
Ribosome biogenesis in eukaryotes	1.508083468	0.03103963	83
Glycerolipid metabolism	1.317040477	0.048190288	44
mRNA surveillance pathway	1.216083193	0.060801852	62
AMPK signaling pathway	1.068082716	0.085490387	70
Fatty acid biosynthesis	1.031002925	0.09311016	35

Table S6 Summary of the KEGG pathways involved in 80 DEGs

term	enrichment	pvalue	count
Caffeine metabolism	0.761695835	0.173102829	1
Glycosaminoglycan degradation	0.744034386	0.180287499	1
FoxO signaling pathway	0.738256246	0.18270219	2
Glutathione metabolism	0.719965845	0.190561058	2
Insect hormone biosynthesis	0.717808581	0.191509984	1
Cutin, suberine and wax biosynthesis	0.701455448	0.19885868	1

Table S7 Summary of the KEGG pathways involved in 941 DEGs

term	enrichment	pvalue	count
Ribosome	24.8713652	1.34E-25	66
Neuroactive ligand-receptor interaction	2.31654719	0.004824506	10
Retinol metabolism	2.052850375	0.008854206	9
Arachidonic acid metabolism	1.140345207	0.072386036	5
Peroxisome	1.137691724	0.072829659	12
ECM-receptor interaction	0.976948642	0.105451159	4

Table S8 Summary of the KEGG pathways involved in 3971 DEGs

term	enrichment	pvalue	count
Fatty acid biosynthesis	4.61807532	2.41E-05	37
AMPK signaling pathway	2.275992189	0.00529673	57
Ribosome biogenesis in eukaryotes	1.515869271	0.030488126	60
Nucleocytoplasmic transport	1.169038736	0.067758107	60
RNA polymerase	1.136407749	0.073045296	27
Cutin, suberine and wax biosynthesis	1.061835822	0.086728968	15

Table S9. Genetic pathways and expression levels TPM

Trehalose Metabolism

Gene_id	Nr_description	Gene Name	L_larvae	S_larvae	L_Female	S_Female
TRINITY_DN22757_c0_g1	alpha,alpha-trehalose-phosphate synthase [UDP-forming] [Chrysoperla carnea]	TPS	27.2933	30.1133	36.3567	37.2367
TRINITY_DN12693_c0_g1	facilitated trehalose transporter Tret1-2 homolog [Chrysoperla carnea]	TRE1	24.8867	24.65	44.33	71.3567
TRINITY_DN3456_c0_g1	facilitated trehalose transporter Tret1-2 homolog [Chrysoperla carnea]	TRE2	24.65	23.51	25.66	45.8167

TRINITY_DN11473_c1_g1	facilitated trehalose transporter Tret1-like [Chrysoperla carnea]	TRE3	24.2433	10.8967	9.12	25.13
TRINITY_DN1315_c2_g1	facilitated trehalose transporter Tret1-like [Chrysoperla carnea]	TRE4	84.8933	78.14	30.2667	23.9133
TRINITY_DN1696_c0_g1	facilitated trehalose transporter Tret1-like [Chrysoperla carnea]	TRE5	9.1867	16.5433	6.3567	7.4367
TRINITY_DN2580_c0_g1	facilitated trehalose transporter Tret1-like [Chrysoperla carnea]	TRE6	22.4467	26.03	38.75	46.98
TRINITY_DN3405_c0_g1	facilitated trehalose transporter Tret1-like [Chrysoperla carnea]	TRE7	23.4	16.9133	6.6833	19.9933

Fatty Acid Metabolism

Gene_id	Nr_description	Gene Name	L_larvae	S_larvae	L_Female	S_Female
TRINITY_DN11122_c0_g2	fatty acid synthase [Chrysoperla carnea]	FAS1	43.9167	27.5767	80.9233	82.37
TRINITY_DN2240_c0_g1	fatty acid synthase [Chrysoperla carnea]	FAS2	13.7933	18.7767	30.5867	25.5333
TRINITY_DN13133_c1_g1	fatty acid synthase-like [Chrysoperla carnea]	FAS3	50.2967	9.94	4.35	0
TRINITY_DN18475_c0_g1	fatty acid synthase-like [Chrysoperla carnea]	FAS4	28.4433	31.1233	19.55	0.27
TRINITY_DN366_c0_g1	acetyl-CoA carboxylase isoform X1 [Chrysoperla carnea]	ACC	25.5867	27.97	33.3133	43.28
TRINITY_DN9587_c0_g1	acyl-CoA Delta-9 desaturase [Chrysoperla carnea]	FAD1	52.3933	58.8567	91.7467	76.67
TRINITY_DN13729_c0_g1	acyl-CoA Delta-9 desaturase-like [Chrysoperla carnea]	FAD2	51.9867	110.7667	111.26	102.2233
TRINITY_DN19300_c0_g1	acyl-CoA Delta-9 desaturase-like [Chrysoperla carnea]	FAD3	6.13	0	18.5033	30.1867
TRINITY_DN2347_c0_g1	acyl-CoA Delta-9 desaturase-like [Chrysoperla carnea]	FAD4	34.9133	10.5	37.8667	164.59
TRINITY_DN2347_c1_g1	acyl-CoA Delta-9 desaturase-like [Chrysoperla carnea]	FAD5	2.6133	0	3.27	43.0433
TRINITY_DN441_c0_g1	acyl-CoA Delta-9 desaturase-like [Chrysoperla carnea]	FAD6	6	1.2133	9.99	50.1467
TRINITY_DN34529_c0_g1	acyl-CoA Delta-9 desaturase-like isoform X2 [Chrysoperla carnea]	FAD7	2.5	0.0567	11.4867	56.9933

TRINITY_DN161_c0_g2	sphingolipid delta(4)	FAD8	10.8833	9.61	18.6633	17.86
TRINITY_DN11624_c0_g1	lipid storage droplets surface-binding protein 1 isoform X2 [Chrysoperla carnea]	LSD1	74.8733	126.4433	160.8833	253.51
TRINITY_DN31336_c0_g1	lipid storage droplets surface-binding protein 2 isoform X2 [Chrysoperla carnea]	LSD2	66.2167	64.3367	133.65	68.53
TRINITY_DN7315_c0_g1	hormone-sensitive lipase [Chrysoperla carnea]	Lipase	1.1267	2.56	3.4367	3.74
TRINITY_DN1996_c5_g2	long-chain fatty acid transport protein 4 [Chrysoperla carnea]	FATP1	4.49	5.89	7.4167	3.35
TRINITY_DN6463_c0_g1	long-chain fatty acid transport protein 4 [Chrysoperla carnea]	FATP2	13.5567	20.67	17.5633	12.97
TRINITY_DN2495_c0_g1	fatty acid-binding protein, heart-like [Chrysoperla carnea]	FABP1	441.5233	1654.7367	0.0367	0
TRINITY_DN1625_c0_g1	fatty acid-binding protein, liver-type-like [Chrysoperla carnea]	FABP2	3721.6333	3397.9867	434.77	500.98
TRINITY_DN453_c0_g1	long-chain-fatty-acid--CoA ligase 1 isoform X1 [Chrysoperla carnea]	ACS1	7.8533	13.98	12.0133	13.1467
TRINITY_DN15243_c0_g1	long-chain-fatty-acid--CoA ligase 4 isoform X1 [Chrysoperla carnea]	ACS2	15.9933	21.0767	29.7467	27.5733
TRINITY_DN317_c0_g1	long-chain-fatty-acid--CoA ligase ACSBG2-like [Chrysoperla carnea]	ACS3	26.4967	28.75	53.74	57.8267
TRINITY_DN6578_c0_g3	carnitine O-acetyltransferase-like [Chrysoperla carnea]	CPT1	13.8133	20.9567	11.5667	13.3033
TRINITY_DN1032_c0_g2	carnitine O-palmitoyltransferase 1, liver isoform isoform X1 [Chrysoperla carnea]	CPT2	3.9567	4.96	8.5167	4.8333
TRINITY_DN12107_c0_g1	carnitine O-palmitoyltransferase 2, mitochondrial [Chrysoperla carnea]	CPT3	3.88	0.9533	4.1467	1.55
TRINITY_DN49490_c0_g1	carnitine O-palmitoyltransferase 2, mitochondrial [Chrysoperla carnea]	CPT4	3.2833	2.4033	3.1667	1.1933
TRINITY_DN702_c0_g2	carnitine O-palmitoyltransferase 2, mitochondrial [Chrysoperla carnea]	CPT5	11.55	12.5333	15.1733	9.24
TRINITY_DN39898_c0_g1	short-chain specific acyl-CoA dehydrogenase, mitochondrial [Chrysoperla carnea]	ACD1	21.0167	34.5233	25.22	21.67
TRINITY_DN2714_c0_g1	short-chain specific acyl-CoA dehydrogenase, mitochondrial-like [Chrysoperla carnea]	ACD2	4.5633	6.0133	4.85	4.4533
TRINITY_DN8646_c0_g1	isovaleryl-CoA dehydrogenase, mitochondrial [Chrysoperla carnea]	ACD3	37.7867	48.1367	7.4967	6.25
TRINITY_DN11696_c0_g2	probable enoyl-CoA hydratase, mitochondrial [Chrysoperla carnea]	ACD4	205.76	357.89	104.31	120.12

TRINITY_DN14742_c0_g2	probable enoyl-CoA hydratase, mitochondrial isoform X1 [Chrysoperla carnea]	ACD5	41.2667	76.76	5.7367	6.2533
TRINITY_DN3489_c0_g1	3-hydroxyacyl-CoA dehydrogenase type-2-like [Chrysoperla carnea]	HCD1	14.95	15.4367	10.9067	9.6433
TRINITY_DN18976_c0_g1	hydroxyacyl-coenzyme A dehydrogenase, mitochondrial-like [Chrysoperla carnea]	HCD2	58.03	100.1633	62.0467	63.8567
TRINITY_DN2959_c0_g1	3-hydroxyisobutyryl-CoA hydrolase, mitochondrial-like isoform X1 [Chrysoperla carnea]	HCD3	31.0967	45.7733	21.1967	18.5067
TRINITY_DN25428_c0_g1	3-ketoacyl-CoA thiolase, mitochondrial [Chrysoperla carnea]	BK	133.7167	208.4867	164.5333	162.6

Pigment Biosynthetic Pathways

Gene_id	Nr_description	Gene Name	L_larvae	S_larvae	L_Female	S_Female
TRINITY_DN9568_c0_g1	4,5-DOPA dioxygenase extradiol-like isoform X1 [Chrysoperla carnea]	DODA	18.62	29.14	13.34	10.9267
TRINITY_DN12781_c0_g1	L-dopachrome tautomerase yellow-f2-like [Chrysoperla carnea]	Dct1	24.81	19.63	30.36	26.0933
TRINITY_DN576_c0_g2	L-dopachrome tautomerase yellow-f2-like [Chrysoperla carnea]	Dct2	30.23	45.5567	5.5	9.9733
TRINITY_DN22162_c0_g1	phenoloxidase 1-like [Chrysoperla carnea]	PO1	24.47	39.3767	3.0067	4.7833
TRINITY_DN56348_c0_g1	phenoloxidase 1-like [Chrysoperla carnea]	PO2	23.02	26.17	2.1933	0
TRINITY_DN7360_c0_g1	phenoloxidase 1-like isoform X1 [Chrysoperla carnea]	PO3	39.3867	48.5367	16.97	13.3033
TRINITY_DN2424_c0_g1	protein yellow-like [Chrysoperla carnea]	yellow1	2.6833	2.2167	8.1833	9.5167
TRINITY_DN25958_c0_g1	protein yellow-like [Chrysoperla carnea]	yellow2	2.87	1.67	3.1933	4.4267
TRINITY_DN22556_c0_g1	laccase-8-like [Chrysoperla carnea]	laccase1	6.52	16.22	0.1767	0.19
TRINITY_DN231_c0_g1	laccase-8-like [Chrysoperla carnea]	laccase2	213.28	248.3	25.0033	21.6267
TRINITY_DN28260_c0_g1	laccase-8-like [Chrysoperla carnea]	laccase3	6.3333	11.5633	0.5633	0
TRINITY_DN40541_c0_g1	laccase-8-like [Chrysoperla carnea]	laccase4	8.44	15.8933	0.0667	0.42

TRINITY_DN53755_c0_g1	laccase-8-like [Chrysoperla carnea]	laccase5	18.33	47.6333	2.5467	4.11
TRINITY_DN53758_c0_g1	laccase-8-like [Chrysoperla carnea]	laccase6	14.17	45.5567	0.77	0.6567
TRINITY_DN8279_c0_g1	laccase-8-like [Chrysoperla carnea]	laccase7	81.83	76.6433	10.5733	10.6533
TRINITY_DN12219_c0_g1	GTP cyclohydrolase 1 isoform X2 [Chrysoperla carnea]	Gch1	67.1067	138.8733	124.38	56.4033
TRINITY_DN1710_c0_g1	arylalkylamine N-acetyltransferase 1-like isoform X1 [Chrysoperla carnea]	aaNAT	1.3067	1.9267	2.0333	1.7033
TRINITY_DN15636_c0_g1	tryptophan 2,3-dioxygenase [Chrysoperla carnea]	TDO	6.34	5.4767	1.24	1.51
TRINITY_DN15805_c0_g1	kynurenine formamidase-like [Chrysoperla carnea]	Kfase1	12.97	13.9733	12.5767	16.75
TRINITY_DN30885_c0_g2	kynurenine formamidase-like [Chrysoperla carnea]	Kfase2	8.5433	9.36	16.12	14.4
TRINITY_DN26062_c0_g1	protein white [Chrysoperla carnea]	white1	0.75	0.5867	5.98	3.0033
TRINITY_DN4325_c0_g1	protein white [Crassostrea gigas]	white2	0.2733	0.01	1.9133	4.26
TRINITY_DN35773_c0_g1	protein scarlet [Chrysoperla carnea]	scarlet1	0.18	0.4067	0.1733	0
TRINITY_DN35773_c0_g3	protein scarlet [Chrysoperla carnea]	scarlet2	0.42	0.4267	0.0633	0.1567
TRINITY_DN417_c8_g1	sepiapterin reductase-like [Chrysoperla carnea]	Spr	11.23	12.2267	24.54	14.92

Ovarian development

Gene_id	Nr_description	Gene Name	L_larvae	S_larvae	L_Female	S_Female
TRINITY_DN18142_c0_g3	vitellogenin [Chrysoperla nipponensis]	Vg	0.0033	0.0167	1888.3933	0.0133
TRINITY_DN7774_c0_g1	vitellogenin receptor [Chrysoperla nipponensis]	VgR	0.1433	0.12	29.4233	6.6433

Juvenile Hormone Pathway

Gene_id	Nr_description	Gene Name	L_larvae	S_larvae	L_Female	S_Female
TRINITY_DN6938_c0_g1	3-hydroxy-3-methylglutaryl-coenzyme A reductase [Chrysoperla carnea]	HMGR	4.2067	4.9	15.4167	7.25
TRINITY_DN7440_c0_g1	mevalonate kinase [Chrysoperla carnea]	MevK	7.9233	8.0633	9.44	7.06
TRINITY_DN1968_c0_g1	probable phosphomevalonate kinase [Chrysoperla carnea]	PMK	5.36	5.3067	5.0233	3.8667
TRINITY_DN5471_c0_g1	diphosphomevalonate decarboxylase isoform X1 [Chrysoperla carnea]	PPMD	3.65	4.3067	5.31	3.7567
TRINITY_DN485_c0_g1	farnesyl pyrophosphate synthase [Chrysoperla carnea]	FPPS	22.7867	34.55	17.81	25.9733
TRINITY_DN20191_c0_g2	farnesyl diphosphate pyrophosphatase [Pardosa pseudoannulata]	FPP	0.01	0	1.0267	0.52
TRINITY_DN11572_c1_g2	farnesol dehydrogenase-like [Chrysoperla carnea]	FOLD1	14.7633	17.5367	40.7333	36.1
TRINITY_DN12303_c0_g1	farnesol dehydrogenase-like [Chrysoperla carnea]	FOLD2	15.69	270.9933	0.0267	0
TRINITY_DN15737_c0_g2	farnesol dehydrogenase-like [Chrysoperla carnea]	FOLD3	50.4867	58.9133	75.8133	102.9467
TRINITY_DN20157_c0_g1	farnesol dehydrogenase-like [Chrysoperla carnea]	FOLD4	25.4867	22.1067	29.2133	31.4433
TRINITY_DN2784_c0_g1	farnesol dehydrogenase-like [Chrysoperla carnea]	FOLD5	37.9333	57.1533	23.1333	22.1067
TRINITY_DN3695_c0_g1	farnesol dehydrogenase-like [Chrysoperla carnea]	FOLD6	39.62	59.4267	126.7	140.9133
TRINITY_DN40785_c0_g1	farnesol dehydrogenase-like [Chrysoperla carnea]	FOLD7	99.96	119.6267	56.4533	74.3067
TRINITY_DN6689_c0_g1	farnesol dehydrogenase-like [Chrysoperla carnea]	FOLD8	110.9267	114.2433	5.2967	6.2067
TRINITY_DN7741_c0_g1	farnesol dehydrogenase-like [Chrysoperla carnea]	FOLD9	200.6567	229.64	9.5	14.31
TRINITY_DN8871_c0_g1	farnesol dehydrogenase-like [Chrysoperla carnea]	FOLD10	5.7433	4.8033	23.5233	25.3633
TRINITY_DN21893_c0_g1	farnesol dehydrogenase-like isoform X2 [Chrysoperla carnea]	FOLD11	159.6967	238.84	142.21	149.6
TRINITY_DN66519_c0_g1	juvenile hormone acid O-methyltransferase-like [Chrysoperla carnea]	Jhamt1	0	0.5	0.2267	0

TRINITY_DN6733_c0_g1	juvenile hormone acid O-methyltransferase-like [Chrysoperla carnea]	Jhamt2	0.6867	2.9	0.83	1.8433
TRINITY_DN11222_c0_g1	juvenile hormone acid O-methyltransferase-like [Chrysoperla carnea]	Jhamt3	2.8667	4.1133	0.3367	0.4233
TRINITY_DN53892_c1_g1	juvenile hormone acid O-methyltransferase-like [Chrysoperla carnea]	Jhamt4	2.6033	3.63	0.9533	0.7567
TRINITY_DN17011_c0_g1	juvenile hormone esterase-like [Chrysoperla carnea]	JHE1	2162.0167	7441.5733	108.5833	30.43
TRINITY_DN376_c0_g1	juvenile hormone esterase-like [Chrysoperla carnea]	JHE2	1331.4067	3391.0767	787.53	386.4367
TRINITY_DN64387_c0_g1	juvenile hormone esterase-like [Chrysoperla carnea]	JHE3	1264.9467	3574.3567	1466.55	644.5033
TRINITY_DN25498_c0_g1	juvenile hormone epoxide hydrolase 1-like isoform X1 [Chrysoperla carnea]	JHEH	41.59	97.2333	29.8567	31.25

Ecdysteroid Pathway

Gene_id	Nr_description	Gene Name	L_larvae	S_larvae	L_Female	S_Female
TRINITY_DN19991_c0_g2	cytochrome P450 307a1-like [Chrysoperla carnea]	Spook1	0	0.21	0.7833	0
TRINITY_DN45471_c0_g2	cytochrome P450 307a1-like [Chrysoperla carnea]	Spook2	0.0633	0	0.44	0
TRINITY_DN38455_c0_g1	cytochrome P450 306a1 [Chrysoperla carnea]	Phantom	0.0567	0.0833	1.23	0.2133
TRINITY_DN11969_c0_g2	cytochrome P450 302a1, mitochondrial-like [Chrysoperla carnea]	Disembodied1	3.36	3.4367	0.0333	0.01
TRINITY_DN31857_c0_g1	cytochrome P450 302a1, mitochondrial-like [Chrysoperla carnea]	Disembodied2	0.1467	0.38	0.3767	0.15
TRINITY_DN10672_c0_g1	cytochrome P450 315a1, mitochondrial [Chrysoperla carnea]	Shadow	1.95	4.32	21.54	12.12
TRINITY_DN7643_c1_g1	ecdysone receptor [Chrysoperla carnea]	EcR	0.1433	0.2367	1.1133	0.5567
TRINITY_DN17054_c0_g1	broad-complex core protein isoform X1 [Chrysoperla carnea]	Br-C1	0.54	1.6533	0.1367	0.05
TRINITY_DN4568_c0_g1	broad-complex core protein isoforms 1/2/3/4/5 isoform X2 [Chrysoperla carnea]	Br-C2	0.4167	0.4633	1.7067	0.91
TRINITY_DN17054_c0_g3	broad-complex core protein isoforms 1/2/3/4/5 isoform X3 [Chrysoperla carnea]	Br-C3	0.7433	0.9133	0.1567	0

TRINITY_DN20840_c0_g1	broad-complex core protein-like isoform X1 [Chrysoperla carnea]	Br-C4	0.8767	1.6633	0.9533	0.9533
TRINITY_DN12825_c0_g1	ecdysone-induced protein 74EF isoform X1 [Chrysoperla carnea]	E74A	0.9833	4.91	1.2167	0.81
TRINITY_DN6000_c0_g1	ecdysone-inducible protein E75 isoform X1 [Chrysoperla carnea]	E75	3.1933	5.5867	5.5933	4.2233
TRINITY_DN23217_c0_g1	Krueppel homolog 1-like [Chrysoperla carnea]	Kr-h1	4.5067	3.93	10.6833	0.3467

Insulin Pathway

Gene_id	Nr_description	Gene Name	L_larvae	S_larvae	L_Female	S_Female
TRINITY_DN21415_c0_g1	insulin receptor substrate 1 isoform X4 [Chrysoperla carnea]	InR1	0.1333	0.3233	1.84	0.5133
TRINITY_DN5561_c0_g1	insulin-like receptor isoform X1 [Chrysoperla carnea]	InR2	0.11	0.23	1	0.2867
TRINITY_DN32751_c0_g1	forkhead box protein O [Chrysoperla carnea]	FoxO	1.4867	1.9667	2.9167	3.08
TRINITY_DN17943_c0_g1	5'-AMP-activated protein kinase subunit beta-1 [Chrysoperla carnea]	AMPK	15.9967	22.0833	24.5	23.7867
TRINITY_DN43526_c0_g1	phosphatidylinositol 4-phosphate 3-kinase C2 domain-containing subunit beta-like [Chrysoperla carnea]	PI3K1	0.41	1.16	0.6967	0.4
TRINITY_DN9406_c0_g1	phosphatidylinositol 4-phosphate 3-kinase C2 domain-containing subunit beta-like [Chrysoperla carnea]	PI3K2	0.2733	0.49	1.64	0.67

Table S10 Major primer sequences in this study

Gene Name	Forward primers	Sequence	Reverse primers	Sequence
TPS	TPS-F	TTTTCGACGTTTCGTCCC	TPS-R	CAGATTTGGCTATTCTTCCG
TRE1	TRE1-F	CAACAGCCATACCCACAC	TRE1-R	GCGTACAAATCGGTTCAAG
FAS2	FAS2-F	CAGGGAATAGATTGCGAC	FAS2-R	AGAAGATTGGACAGTGGCT
FAD1	FAD1-F	CCAGTTGCAGCATTCTCT	FAD1-R	GGCCACTGGATTACGTAC

ACS2	ACS2-F	TCAACACCGTAGCATCACC	ACS2-R	GCTTCTTACCATTGGCACA
ACD1	ACD1-F	AGGAGCAGCATCAACTATG	ACD1-R	CCTTCACTTTCGTAACCG
HCD2	HCD2-F	TGGGTTCAGGAATTGCAC	HCD2-R	CACCATCTTGTGGCTTATCT
BK	BK-F	ACTAATCGGGCAAGTGGT	BK-R	GGCTTCTGGAATCTGTGA
PO1	PO1-F	ATTGTGGTTGTGGATGGC	PO1-R	GACGAATCTGAACACGGAC
yellow1	yellow1-F	CCACTGCTCGGGTCTTAT	yellow1-R	GCACCATGTATTTCCATCC
Jhamt1	Jhamt1-F	CCAAGTTTAGAAATTGATTCATATAT	Jhamt1-R	TTGAAACCAAAAGGTGATGTG
Jhamt2	Jhamt2-F	CGGTAAATCCATTCCTGAATCG	Jhamt2-R	GCGTACACAATAAGTTCGTCAT
Jhamt3	Jhamt3-F	TTACCGCACCATCTCCGCA	Jhamt3-R	CGCTTCAGTAAACGTGGTAACAAG
Jhamt4	Jhamt4-F	AACGATTGTTGGCACAGATC	Jhamt4-R	GGCAAAGTTTTTACCCCG
JHE1	JHE1-F	ATGGCTCAGGTGGTTGGGGT	JHE1-R	GGCGTATTGCTAAAGATGCGAA
JHEH	JHEH-F	TGCAAAATGTCCGCCTCT	JHEH-R	CCAACAAATGTTCCATCAGC
EcR	EcR-F	GATGACTGGCTAACAACGA	EcR-R	CTGTAAACGAATGTCCTGG
Kr-h1	Kr-h1-F	ATACCGGCGAGAAACCGT	Kr-h1-R	GACCATAATGTGCCACTTGA
InR1	InR1-F	AAGGTCCACAGTCAGCAGG	InR1-R	TTTGATGGAGGACTTGCG
Jhamt1	dsJhamt1-T7-F	taatacgactcactataggATTAAATCCTCGTAAATAAATGTTC	dsJhamt1-T7-R	taatacgactcactataggTTAGTTACATATCTCGCAAGTAATCC
	dsJhamt1-F	ATTAAATCCTCGTAAATAAATGTTC	dsJhamt1-R	TTAGTTACATATCTCGCAAGTAATCC
GFP	dsGFP-T7-F	taatacgactcactataggTGACCCTGAAGTTCATCTG	dsGFP-T7-R	taatacgactcactataggGGTGTTCTGCTGGTAGTG
	dsGFP-F	TGACCCTGAAGTTCATCTG	dsGFP-R	GGTGTTCTGCTGGTAGTG
